# Circulating MicroRNA Profiling in Non-ST Elevated Coronary Artery Syndrome Highlights Genomic Associations with Serial Platelet Reactivity Measurements

**DOI:** 10.1038/s41598-020-63263-6

**Published:** 2020-04-10

**Authors:** Kristian C. Becker, Lydia Coulter Kwee, Megan L. Neely, Elizabeth Grass, Joseph A. Jakubowski, Keith A. A. Fox, Harvey D. White, Simon G. Gregory, Paul A. Gurbel, Leonardo de Pinto Carvalho, Richard C. Becker, E. Magnus Ohman, Matthew T. Roe, Svati H. Shah, Mark Y. Chan

**Affiliations:** 1 Icahn School of Medicine at Mount Sinai, New York, NY USA; 2 Duke Molecular Physiology Institute, Durham, NC USA; 3 Duke Clinical Research Institute, Durham, NC USA; 4 Division of Cardiology, Duke University School of Medicine, Durham, NC USA; 5 Eli Lilly and Company, Indianapolis, IN USA; 6 University of Edinburgh, Edinburgh, UK; 7 Green Lane Cardiovascular Service, Auckland City Hospital, Auckland, New Zealand; 8 Inova Heart & Vascular Institute, Falls Church, VA USA; 9 Albert Einstein Hospital, Sao Paolo, Brasil; 10 University of Cincinnati, Cincinnati, Ohio USA; 11 National University of Singapore, Singapore, Singapore

**Keywords:** Clinical genetics, Platelets, miRNAs

## Abstract

Changes in platelet physiology are associated with simultaneous changes in microRNA concentrations, suggesting a role for microRNA in platelet regulation. Here we investigated potential associations between microRNA and platelet reactivity (PR), a marker of platelet function, in two cohorts following a non-ST elevation acute coronary syndrome (NSTE-ACS) event. First, non-targeted microRNA concentrations and PR were compared in a case (N = 77) control (N = 76) cohort within the larger TRILOGY-ACS trial. MicroRNA significant in this analysis plus CVD-associated microRNAs from the literature were then quantified by targeted rt-PCR in the complete TRILOGY-ACS cohort (N = 878) and compared with matched PR samples. Finally, microRNA significant in the non-targeted & targeted analyses were verified in an independent post NSTE-ACS cohort (N = 96). From the non-targeted analysis, 14 microRNAs were associated with PR (Fold Change: 0.91–1.27, p-value: 0.004–0.05). From the targeted analysis, five microRNAs were associated with PR (Beta: −0.09–0.22, p-value: 0.004–0.05). Of the 19 significant microRNAs, three, miR-15b-5p, miR-93 and miR-126, were consistently associated with PR in the TRILOGY-ACS and independent Singapore post-ACS cohorts, suggesting the measurement of circulating microRNA concentrations may report on dynamic changes in platelet biology following a cardiovascular ischemic event.

## Introduction

Despite advancements in its management, cardiovascular disease including non-ST elevation acute coronary syndrome (NSTE-ACS) remains a major cause of patient morbidity and mortality^1–4^. Antiplatelet therapies including P2Y_12_ antagonists are used to treat NSTE-ACS acutely and in the longer-term. While measurements of platelet reactivity (PR) have facilitated the rapid quantification of anti-platelet medication efficacy, questions still remain about the utility of PR to predict clinical outcomes^5–8^. Platelet genetics and PR sub-studies from large-scale clinical trials have informed our understanding of individual variability in P2Y_12_ inhibitor response, but genomic regulation of individual patient’s PR has not been fully clarified^9–14^.

Small non-coding ribonucleic acids (RNA) including microRNAs have emerged as potential regulators of PR in patients with cardiovascular disease^15^. MicroRNAs (miRNAs) are 18–22 nucleotide non-coding RNAs that play an essential role in gene modulation and expression and are established regulators of cardiovascular pathology^16–19^. Platelets have been shown to be an important source of circulating miRNAs^19–21^. However, the association between specific circulating miRNA levels and PR has not been previously studied in a large NSTE-ACS population. Additionally, the changes in miRNA concentrations and PR over the first 6 months following a cardiovascular event have not been studied, and an investigation of these changes may lead to better understanding of the molecular mechanisms underlying dynamic changes in PR that occur in the weeks to months following a NSTE-ACS event and any ensuing intervention such as cardiac catheterization and stenting^22,23^.

Therefore, to better understand the relationship between changing microRNA levels and PR following a NSTE-ACS event we profiled circulating miRNAs and PR in patients before and during treatment with one of two P2Y_12_ antagonists, clopidogrel or prasugrel, within the framework of a large randomized controlled trial, the Targeted Platelet Inhibition to Clarify the Optimal Strategy to Medically Manage Acute Coronary Syndromes (TRILOGY-ACS) trial. Within the TRILOGY-ACS cohort we first chose a case/control cohort to perform an initial exploratory analysis using non-targeted miRNA sequencing to discover significant miRNA associated with PR. A targeted analysis was then conducted in the complete TRILOGY-ACS cohort focusing on the miRNA significant in the non-targeted cohort plus miRNAs from the literature known to be associated with inflammation and cardiovascular disease. We then compared our findings against an independent NSTE-ACS cohort in Singapore to verify that our results applied to a wide range of patients and demographics. We hypothesized that large-scale serial miRNA profiling identifies a set of circulating miRNAs that associate with PR in patients with NSTE-ACS and that these miRNA-PR associations evolve over time following an NSTE-ACS event.

## Results

### Patient characteristics

**Figure 1:**
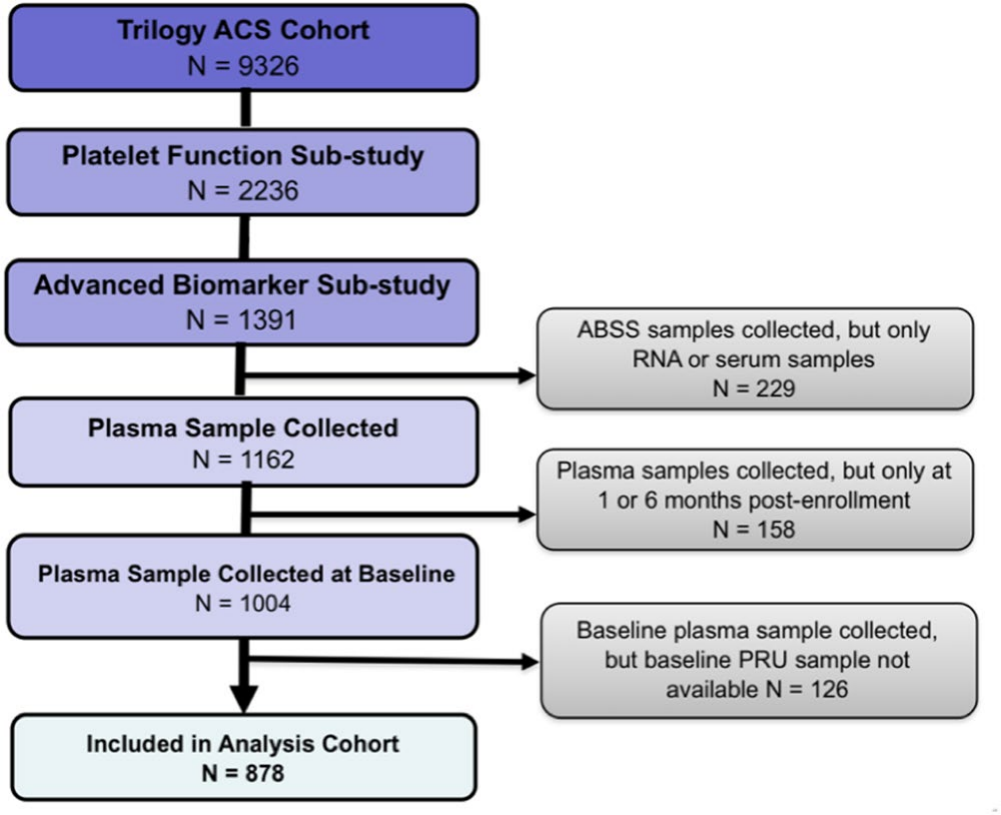
TRILOGY-ACS analysis cohort. Our primary analysis cohort was derived from patients included in the TRILOGY-ACS and subsequent Advanced Biomarker Sub-study. The primary TRILOGY-ACS trial found no difference in outcomes (CV death/MI/stroke) between prasugrel vs. clopidogrel treatment groups. Further details of the TRILOGY-ACS and the Advanced Biomarker Sub-study are included in the Supplemental Methods.

The primary analysis cohort consisted of 878 individuals from within the TRILOGY-ACS trail and its Advanced Biomarker Sub-study. Selection details are included in Fig. 1.

At baseline, there were differences in race between the overall TRILOGY-ACS clinical trial and ABSS sub-study, but there were no significant differences between the ABSS and the final analysis cohort (Table 1).

**Table 1:** Baseline characteristics of the TRILOGY-ACS study populations.

	TRILOGY-ACS	Subgroup with PRU and blood samples from TRILOGY-ACS	Targeted Analysis Cohort	Nested Case-Control Cohort
**Demographic Information**	N = 9326	N = 1391	N = 878	N = 153
Age (years)	65.7 (59.0, 74.0)	66.0 (59.0, 74.0)	67.0 (59.0, 73.0)	70.0 (64.0, 76.0)
Male, N (%)	5676 (60.9%)	859 (61.8%)	541 (61.6%)	106 (69.3%)
White, N (%)	6276 (67.3%)	1141 (82.0%)	725 (82.6%)	127 (83.0%)
BMI, kg m^−2^	27.1 (24.2, 30.5)	27.7 (25.0, 31.1)	27.6 (25.1, 31.1)	27.8 (24.8, 31.1)
**Cardiovascular Risk Factors**	2840 (31.7%)	438 (32.4%)	221 (32.1%)	221 (32.1%)
Cr Cl (ml/min)	73.0 (54.0, 96.5)	75.9 (56.0, 100)	75.4 (56.2, 100)	65.9 (51.1, 90.5)
Current or recent smoker, N (%)	1715 (18.6%)	258 (18.8%)	155 (17.8%)	31 (20.7%)
Diabetes mellitus, N (%)	3539 (38.0%)	500 (36.0%)	313 (35.7%)	56 (36.8%)
GRACE risk score	121.0 (105.0, 139.0)	124.0 (106.0, 144.0)	124.0 (108.0, 143.3)	133.0 (116.5, 154.0)
Prior MI, N (%)	3987 (43.1%)	652 (47.1%)	392 (44.9%)	72 (47.4%)
**Concomitant Medications**				
Aspirin, N (%)	8572 (91.9%)	1280 (92.0%)	817 (93.1%)	144 (94.1%)
Beta blocker, N (%)	7251 (77.8%)	1118 (80.4%)	705 (80.3%)	121 (79.1%)
Statin, N (%)	7776 (83.4%)	1164 (83.7%)	746 (85.0%)	134 (87.6%)
Assigned to prasugrel arm, N (%)	4663 (50.0%)	690 (49.6%)	418 (47.6%)	74 (48.4%)
Index NSTEMI, N (%)	6520 (69.9%)	938 (67.4%)	591 (67.3%)	135 (88.2%)
Index Unstable angina, N (%)	2302 (24.7%)	366 (26.3%)	231 (26.3%)	14 (9.2%)
CVD/MI/Stroke within 1 yr. of index event, N (%)	954 (10.2%)	156 (11.2%)	81 (9.2%)	77 (50.3%)

Continuous variables are presented as median (25^th^ and 75^th^ percentile) and categorical variables are presented as percentages.

Baseline characteristics of the independent Singapore cohorts A and B are included in the Supplemental Materials. In brief, Singapore cohorts A (N = 48) and B (N = 48) showed no significant differences in age, sex, race, BMI, diabetes prevalence, coronary artery disease or smoking prevalence between the HPR group and the LPR groups (Suppl. Table 3A,B). The Singapore cohort mainly consisted of continental Asian patients (Chinese, Malay and South Asian) while the TRILOGY-ACS cohort consisted of mainly Caucasian, Latino and African American patients. Age, sex and BMI characteristics were not significantly different between the Singapore and TRILOGY-ACS cohorts.

### MiR-Seq profiling and baseline platelet reactivity in the TRILOGY-ACS cohort

Of the 247 miRNAs expressed and identified in whole blood samples prior to anti-platelet medication administration (baseline) by non-targeted miR-Seq analysis, 14 were associated with PR including: miR-10a-5p, miR-15b-5p, miR-21-3p, miR-25-5p, miR-93-3p, miR-197-3p, miR-324-5p, miR-345-5p, miR-589-5p, miR-671-3p, miR-939-5p, miR-1294, miR-3609, and miR-4732-3p (Table 2). MiRNA Log_2_ Fold Change ranged from: −0.13 to 0.35, with p-values from 0.005 to 0.05. Overall, miR-345-5p demonstrated the strongest association (Log_2_ FC: −0.11, P = 0.005), with lower miR-345-5p values associated with higher PR values.

**Table 2:** Fourteen miRNAs are associated with platelet reactivity in the baseline whole blood TRILOGY cohort by non-targeted miR-Seq analysis. Fold Changes represent Log_2_ Fold Changes in miRNA concentration per 1 SD PRU unit. Fold changes ranged from −0.12–0.35 with p-values from 0.004–0.05. MiRNA species are listed from smallest to largest p-value. € MiRNA species not included in the Singapore Nanostring miRNA analysis set.

miRNA	Log_2_ Fold Change	P-value
miR-345-5p	−0.11	0.005
miR-197-3p	0.16	0.01
miR-939-5p	0.28	0.01
miR-15b-5p	0.13	0.03
miR-93-3p	0.09	0.03
miR-1294€	0.13	0.04
miR-589-5p	−0.12	0.04
miR-25-5p	0.23	0.04
miR-21-3p	−0.12	0.04
miR-324-5p	−0.14	0.05
miR-671-3p	0.18	0.05
miR-4732-3p€	0.11	0.05
miR-10a-5p	0.21	0.05
miR-3609€	0.35	0.05

### Serial targeted rt-PCR profiling and platelet reactivity in the TRILOGY-ACS cohort

From the targeted plasma miRNA array, five of the 46 analyzed miRNA species were associated with PR by Generalized Estimating equations (GEE) analysis in the combined 30-day & six-month time-points: miR-574-3p (Beta: −0.1, P = 0.006), miR-345-5p (Beta: −0.22, P = 0.02), miR-126-5p (Beta: −0.08, P = 0.03), Let-7d-3p (Beta: −0.08, P = 0.031) and miR-191-5p (Beta: 0.08, P = 0.049).

**Figure 2:**
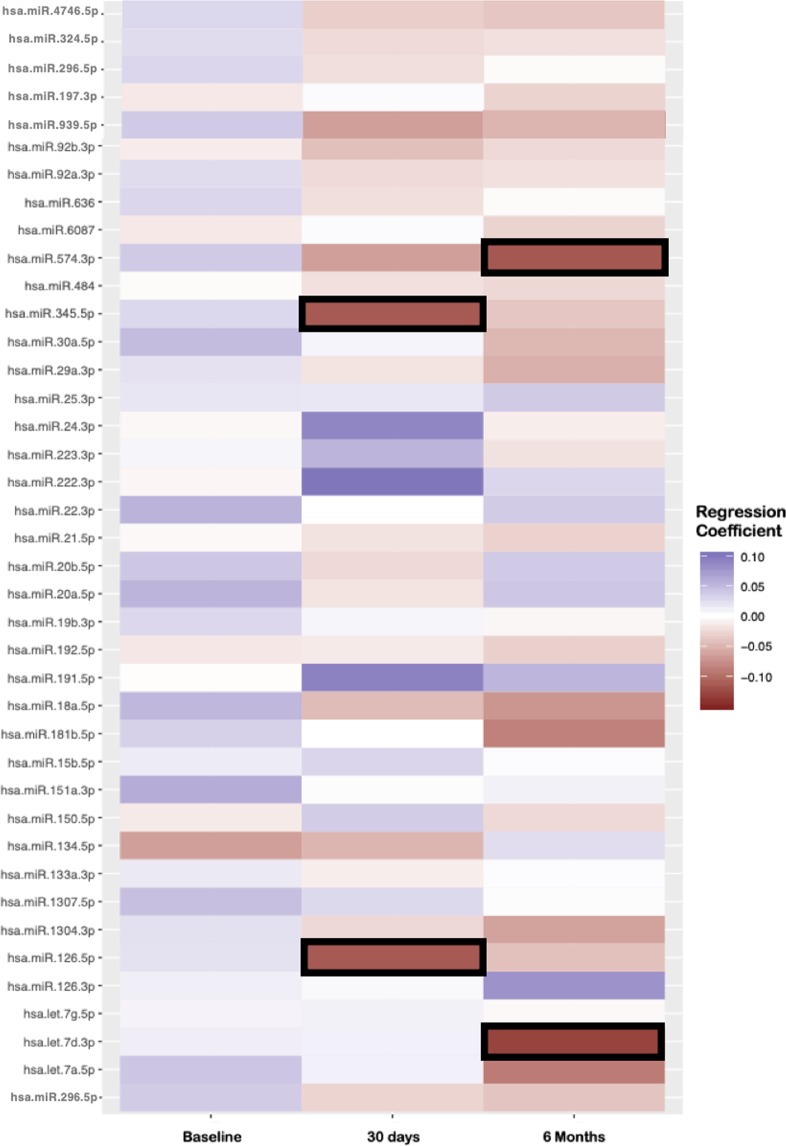
MiRNAs associated with platelet reactivity in the TRILOGY-ACS cohort by serial real-time PCR array. A heatmap of miRNA expression levels against platelet reactivity units (PRU) at three distinct timepoints during the TRILOGY-ACS trial; baseline, 30 days and 6 months post-CVD event on P2Y_12_ anti-platelet therapy. Bolded coefficients represent miRNA with p-values < 0.05. Regression analysis was performed using miRNA/PR data within individual time-points and was adjusted for clinical covariates.

In a multivariable regression analysis of the five significant miRNA species‚ four of the five were associated with PR in at least one individual time point. No miRNA species were associated with PR at the baseline time-point. MiR-126-5p (N = 464, Beta: −0.14, P = 0.013) and miR-345-5p (N = 462, Beta: −0.29, P = 0.03) were associated with PR at the 30-day time point. MiR-574-3p (N = 435, Beta: −0.13, P = 0.02) and Let-7d-3p (N = 427, Beta: −0.16, P = 0.004) were associated with PR at the six-month time point (Fig. 2).

### Comparison with miRNAs in the independent Singapore cohort

Of the 800 miRNAs within the Singapore cohort, 23 miRNAs were significantly associated with PR in either cohort A or B (Suppl. Table 4). The Singapore analysis had comprehensive overlap with the TRILOGY cohort analyses; of the 14 miRNAs associated with PR in the miR-Seq analysis three (miR-1294, miR-4732-3p and miR-3609) were not included in the Singapore analysis. Importantly, all five miRNAs significantly associated with PR in the targeted rt-PCR analysis were included.

**Figure 3:**
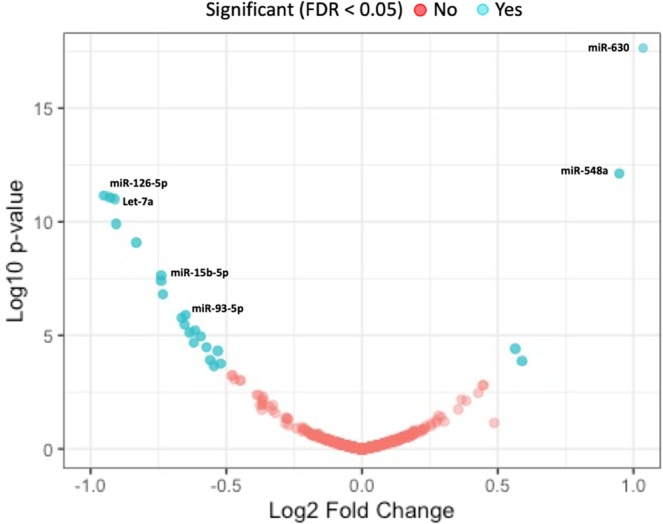
Plasma miRNA Associated with Platelet Reactivity within the independent Singapore cohort. Volcano plot of 800 miRNA concentrations measured by Nanostring compared against platelet reactivity measured by VASP (cohort A) and Multiplate ADP (cohort B). Blue dots represent significantly associated miRNA at FDR < 0.05.

Of the 14 miRNAs significantly associated with PR in the non-targeted miR-Seq analysis, miR-15b-5p and miR-93 were also associated with PR in the Singapore cohort. MiR-15b-5p cohort (cohort A, P = 0.06 and cohort B, P = 2.0 × 10^−11^) was shown to be strand specific and directionally consistent between the TRILOGY-ACS and Singapore cohorts. Of note, miR-93-3p was significant in the TRILOGY-ACS cohort (P = 0.03) while miR-93-5p was significant in the Singapore cohort (cohort A, P = 0.01 and cohort B, P = 0.0004). Of the five miRNAs associated with PR in the targeted miRNA analysis, only miR-126 also showed an association with PR in the Singapore cohort. From the TRILOGY-ACS cohort miR-126-5p was correlated with PR (P = 0.012) while miR-126-3p was correlated with PR in the Singapore cohort (cohort A, P = 9 × 10^−3^ and cohort B, P = 3.2 × 10^−11^) (Fig. 3).

**Figure 4:**
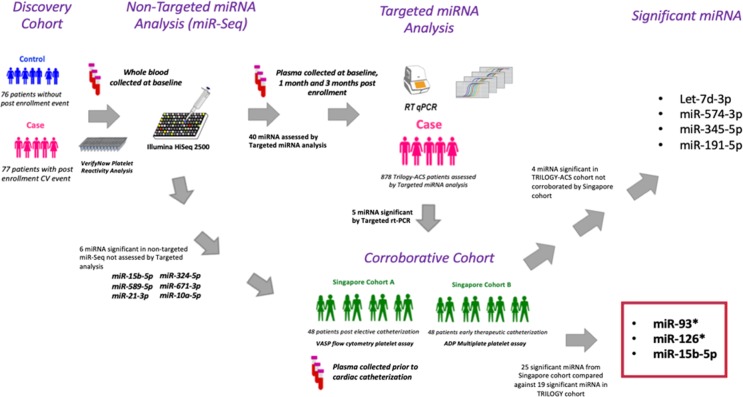
Visual Abstract of whole blood miRNA profiling by miR-Seq and plasma miRNA profiling by miRNA rt-PCR array in the TRILOGY-ACS cohort and plasma profiling by Nanostring in the Singapore cohorts. Samples in the whole blood TRILOGY cohort (N = 153) and the plasma Singapore cohorts (N = 96) were collected following 7 days of maintenance clopidogrel (Baseline). Platelet-poor plasma samples for the TRILOGY cohort targeted array were collected at Baseline (N = 878), at 30 days of either clopidogrel or prasugrel anti-platelet therapy (N = 466), or at 6 months of clopidogrel or prasugrel therapy (N = 435). *: −3p or −5p miRNA strand.

Overall, miR-93, miR-126 and miR-15b-5p were shown to be significant in both the TRILOGY-ACS cohort and the independent Singapore cohort. Figure 4 further details and summarizes the experimental design, cohorts, sample types, miRNA/platelet assay measurements and significant results within our study.

## Discussion

Using multi-platform miRNA profiling and simultaneous platelet reactivity (PR) measurements from the TRILOGY-ACS trial and a corroborative NSTE-ACS cohort, we identified several circulating miRNA species associated with PR. Our primary investigation included both a non-targeted miR-Seq analysis and a targeted serial miRNA rt-PCR analysis at three distinct time-points after a cardiovascular event (baseline, 3 months, 6 months) in order to understand the dynamic relationship between circulating miRNA and PR in the NSTE-ACS patient. The non-targeted analysis was used as a discovery cohort to determine how a NSTE-ACS event may impact miRNA concentrations and associated PR directly, and as a means of identifying potential highly-altered miRNA to include in the targeted array. The targeted array was then utilized to better understand how miRNA and PR may change over the first 6 months following a pro-inflammatory ischemic event as the body reacts and recuperates at the molecular level.

Three miRNAs, miR-15b-5p, miR-93 and miR-126 were associated with PR in both the TRILOGY-ACS and the Singapore post-ACS cohort. Between the TRILOGY-ACS and Singapore cohorts one miRNA, miR-15b-5p, was found to be both strand specific (either 3p or 5p, denoting the complementary mRNA strand the miRNA binds to and inhibits) and directionally consistent (miRNA concentration associated with increased or decreased PR) across both. These results are consistent with previous data suggesting miR-15b-5p modulates PR by targeting *SENP5*, a SUMO isopeptidase that is activated in the context of platelet activation, ischemia-reperfusion injury and cardiomyopathy^24–26^.

Importantly, and unique to our study design, we also identified five miRNAs (Let-7d-3p, miR-126-5p, miR-191-5p, miR-345-5p and miR-574-3p) that are associated with PR at distinct time points following a NSTE-ACS event. In our analysis, miR-126-5p and miR-345-5p were significantly associated with PR at the 30-day post-ACS event time point and miR-574-3p and Let-7d-3p were significantly associated with PR at the six-month time point. MiR-191-5p demonstrated significant association with PR in the combined 30-day and six-month analysis but did not retain significance in either individual time point cohort. The lack of overlap between the TRILOGY-ACS non-targeted and the targeted cohort analyses may represent this time-dependent change of miRNA when compared to PR, with the non-targeted cohort representing baseline miRNA genomic physiology, and the targeted cohort representing post NSTE-ACS event physiology (30 days and 6 months post NSTE-ACS)^27^.

### MiR-126-5p, miR-345-5p, miR-574-3p and Let-7d-3p are associated with platelet reactivity following NSTE-ACS across time points

Previous literature has noted miR-126 as a highly stable miRNA associated with platelet physiology and a possible novel platelet biomarker in the NSTE-ACS population^27^. While miR-126-5p was not associated with PR in the Singapore cohorts, the 3’ strand of pre-miR-126 (miR-126-3p) was significantly associated with PR. Importantly, miR-126 has been shown to correlate with PR in patients with ACS and is likely a strong marker of platelet function^15^. MiR-126-5p promotes endothelial proliferation and endothelial cell stability and has been implicated in protective properties including limiting atherosclerosis by suppressing Notch1 inhibitor delta-like 1 homolog and reducing thrombogenicity in diabetes mellitus by targeting tissue factor^28–30^. It is therefore credible that lower PR (i.e. lower platelet aggregation), which is associated with fewer ischemic events after ACS, would be associated with higher levels of miR-126-5p, and be consistent with the results we observed in the TRILOGY-ACS cohort. In contrast, miR-126-3p, the 3’ strand of pre-miR-126 was positively correlated with PR in the Singapore cohorts. Both the 5’ and 3’ strand of miR-126 have been shown to be functional in platelet and vascular biology, with miR-126-3p showing significant effects in platelet activation and coagulation^31,32^, while miR-126-5p showing activity in regulating endothelial inflammation and promoting vascular health^33–35^. It is likely that both miR-126-3p and miR-126-5p play a role in post-ACS inflammatory regulation at the platelet and endovascular levels respectively, and additional functional work will be needed to delineate the modes and locations of miR-126 modulation within this dynamic time period following an ischemic event.

MiR-345-5p has not been previously associated with platelet biology and little is known about its function. Mir-345-3p however has been recently shown to increase in concentration in early heart failure, attenuates LDL apoptosis induced inflammation and is a known modulator of insulin like growth factor receptor-1 (IGF-1), which is itself a strong potentiator of platelet activation^36–40^. The association of Let-7d-3p and miR-574-3p with PR was observed 6 months after the index NSTE-ACS event. Let-7d-3p belongs to the Let-7 superfamily and has been previously shown to be an important inducer of vascular smooth muscle cell proliferation via K-RAS^41–43^. MiR-574-3p has been shown to be a marker of inflammation and vascular damage in patients with Type 2 Diabetes, as well as a promoter of vascular smooth muscle growth and a potential novel biomarker of coronary artery disease progression^44–46^.

Overall, this study represents the largest analysis of the association between circulating miRNAs and PR, with results proposing several unique miRNA species as new markers of PR in the context of NSTE-ACS. This study supports the hypothesis that the genomics of circulating miRNA are sensitive to systemic environmental changes such as post-ACS inflammatory conditions and that the molecular physiology of PR is dynamically altered following an NSTE-ACS event.

### Study limitations

Our study is the largest known study to correlate circulating miRNAs with PR data, and the largest with serial patient miRNA samples, however some limitations were present. First, a number of patients within the TRILOGY-ACS were exposed to heparin prior to sample collection. Heparin has been shown to affect the detection of extracellular miRNA concentrations 60 minutes after administration, but its effects on platelet-derived miRNA are not known^47–50^. Our analysis was adjusted for heparin use at the time of sample collection in order to eliminate this potential confounder. Furthermore, study participants were not exposed to heparin at the 30-day and six-month time-point sample collections, and patients in the Singapore cohorts were not exposed to heparin prior to sample collection. A potential limitation of our study was the use of multiple PR measurement modalities; with the TRILOGY-ACS cohort using the VerifyNow ADP assay, the Singapore A cohort using the VASP PRI assay and the Singapore B cohort using the Multiplate ADP test assay. Each method was verified against internal controls during our study, and each method has been shown to closely correlate with each other in patients with ACS with all methods able to discriminate between high and low PR and positively predict major clinical outcomes in multiple individual studies and meta-analyses^51–53^. Additionally, we believe that the corroborated associations between miRNA concentrations and PR across multiple clinically-validated assays emphasizes our results, suggesting the changes in each miRNA following an ACS event are valid as the results remained consistent across different PR assays. The size of the Singapore corroboration cohort was limited to 96 patients and represents a possible limitation in the validation of miRNAs from the TRILOGY-ACS cohort. The Singapore cohort demonstrated internal consistency between cohorts A and B and presents results that are highly correlated with PR measurements (i.e. highly statistically significant) with its cohort size being comparable to other global miRNA profiling datasets^54,55^. Finally, the magnitude of changes in PR mediated by miRNAs was shown to be modest in both the TRILOGY-ACS and Singapore cohorts; this is not unexpected, as miRNAs represent an upstream modulation of protein translation with multiple downstream targets and thus may represent only a modest effect on PR within a landscape of multiple biological mediators^56,57^. More investigation and functional analysis would be required to determine the specific size of downstream effects and the impact of those effects on clinical outcomes.

## Conclusions

This is the largest known study to date of simultaneous microRNA and PR profiling in an NSTE-ACS cohort. Our analysis of microRNA and PR data from the TRILOGY-ACS cohort, a large-scale NSTE-ACS cohort, and the independent Singapore cohort, a delineated NSTE-ACS catheterization cohort, presents an expanded picture of miRNA and its changing association with PR over time following an ischemic event. These findings support a previously proposed pathway of miR-15b-5p’s regulation of PR in patients with ischemic injury and may therefore serve as a biomarker of changes in platelet biology following an NSTE-ACS event. Furthermore, we identified multiple additional miRNA species that are associated with PR at distinct time points, three & six months following an ACS event, suggesting ongoing changes in the genomic architecture regulating PR in a post-ACS context. Through this and further research, microRNA profiling may find a role in monitoring P2Y_12_ drug efficacy in patients with ACS.

## Methods

### Study populations

#### TRILOGY-ACS cohorts

The patient eligibility criteria, trial design, results and analyses of the TRILOGY-ACS and TRILOGY Platelet Function sub-study have been previously reported (NCT00699998)^6,58^. In brief, the TRILOGY-ACS trial was a randomized control trial including 9,326 patients at 800 international trial sites with NSTE-ACS under medical treatment who were randomized to one of two long-term antiplatelet therapies (clopidogrel 75 mg daily or prasugrel 10 mg daily) with all data collection, storage and protection performed by Eli Lilly. The TRILOGY-ACS met guidelines outlined within the Declaration of Helsinki, and conformed to national and local regulatory guidelines detailed within the trial protocol in all participating countries and at all sites. Informed consent was provided and confirmed for all participants according to guidelines at each individual trial site. Overall, the TRILOGY-ACS trial concluded that there was no difference in CV death, myocardial infarction or stroke between prasugrel and clopidogrel over 36 months (2008–2011). Following the initial trial, a sub-section of TRILOGY-ACS patients were then enrolled in secondary sub-studies including a platelet function sub-study (N = 2564) and Advanced Biomarker sub-study (TRILOGY-ABSS) (N = 1391). Patients enrolled in these sub-studies provided whole blood samples at three timepoints including at a baseline time point (prior to P2Y_12_ antiplatelet medication randomization), at 30 days and at six months of clopidogrel or prasugrel medication administration. ABSS participants also provided platelet-poor plasma samples for determining miRNA concentrations at the three time points of baseline, 30 days, and six months. In these sub-studies, PR was measured using the VerifyNow (Accriva Diagnostics, Bedford, USA) P2Y_12_ assay, with PR expressed as P2Y_12_ Units (PRU)^6^. Prior to administration of study drug, PR data of the VerifyNow P2Y_12_ assay was normally distributed. Initial study design, rational and protocols were approved by the Ethical Review Board of the Duke University School of Medicine^58^. Ethical review and oversight throughout the study were conducted by Eli-Lilly Corporation for all 966 participating clinical sites. Each site received approval from their respective ethics review boards and all study participants gave written informed consent prior to study participation. Further detailed information including subject eligibility criteria, sample collection methods and PR profiling measurements for the TRILOGY-ACS and Singapore cohorts are included in the Supplemental Methods.

To determine an association between PR and miRNA concentrations we utilized two distinct cohorts from within the TRILOGY-ACS population. The first cohort was comprised of a nested case-control population with cases defined as subjects with a major adverse cardiovascular event (CV death, MI or stroke) within one year after enrollment in TRILOGY-ACS, and controls were defined as patients without an event during the same follow-up period. Cases and controls were matched for demographics. Cases and controls provided whole blood at baseline, with non-targeted miRNA sequencing (miR-Seq) data and PR measurements performed for analysis. The second cohort included patients from the TRILOGY-ABSS cohort with targeted miRNA PCR profiling and concomitant PR measurements in platelet-poor plasma at any of three time-points (baseline, 30 days or six months). All subjects in both the non-targeted Case-Control cohort and the targeted ABSS cohorts were required to have non-missing *CYP2C19* genotype data, a known effector of P2Y_12_ metabolism, in order to be included in the analysis^59^.

#### Independent Singapore cohort

PR and miRNA profiling were performed in two nested case-control populations collected at the National Hospital in Singapore. In cohort A, platelet-poor plasma samples were collected from 125 patients undergoing elective diagnostic cardiac catheterization at the National University Heart Centre (NUHC), Singapore. From the 125 patients, twenty-four patients with a vasodilator stimulated phosphoprotein phosphorylation assay (Diagnostica Stago, Parsippany, USA) platelet reactivity index (VASP PRI) ≥ 50% (high on-treatment platelet reactivity, HPR) were matched to 24 patients with a VASP PRI < 50% (low on-treatment platelet reactivity, LPR). In cohort B, whole blood samples were drawn from 198 patients with NSTE-ACS undergoing early angiography as part of the multi-center Stratifying risk with Metabolomics And platelet Reactivity Testing in ACS (SMART-ACS) study (NUHC, Singapore)^60^. Cohort B PR values were measured using the Multiplate ADP test assay (Roche Diagnostics, Basel, Switzerland). From the 198 patients in cohort B, twenty-four patients with ADP > 46 aggregation units (AU) (HPR) were matched with 24 patients with ADP < 46 AU (LPR). All patients were pre-treated with a 300 mg loading dose and a 75 mg daily dose of clopidogrel prior to cardiac catheterization and did not receive heparin for at least 24 hours prior to blood sample collection. The study enrollment and protocol of both Singapore population studies were approved by the Singapore National Healthcare Group Domain Specific Review Board and all participants gave written informed consent prior to study participation. Further information regarding PR profiling in the Singapore cohorts are detailed in the Supplemental Materials.

#### MiRNA prioritization

Non-targeted miR-Seq was performed in baseline frozen whole blood samples from the nested incident event case-control TRILOGY-ACS cohort (Fig. 4). MiRNA species found to be significantly associated with PR values were then assessed in the serial TRILOGY-ABSS plasma cohort using targeted miRNA rt-PCR profiling. MiRNA species investigated in the rt-PCR targeted array were chosen based on three potential criteria; 1.) High concentration miRNAs associated with recurrent CVD events in the miR-Seq analysis (N = 30 miRNAs), 2.) the top 5 miRNAs significantly associated with PR in the non-targeted miR-Seq analysis (N = 5), and 3.) miRNAs determined to have association with potential CVD phenotypes from the literature (N = 11 miRNAs) (Suppl. Tables 1 & 2). Finally, miRNA determined to be associated with PR by non-targeted or targeted profiling in the TRILOGY-ACS cohort were corroborated in the Singapore cohort using targeted miRNA profiling.

### MiRNA concentration profiling

#### TRILOGY-ACS non-targeted miRNA sequencing (miR-Seq)

Total whole blood RNA contents were extracted using the PerfectPure RNA blood kit (5Prime, Gaithersburg, MD). MiRNA libraries were prepared using TruSeq prep-kits (Illumina, San Diego, USA). Prior to miRNA library pooling, a Bioanalyzer DNA1000 chip (Agilent, Cary, USA) was used for library size validation and libraries were then quantified using the KAPA Library Quantification kit. MicroRNA pooled-library sequencing was performed on an Illumina HiSeq. 2500 as single-end 50 bp sequence runs using rapid run flow cells. Raw miRNA sequence reads were processed using c*utadapt* v1.5 to remove Illumina sequencing adapters and low quality 3’ sequence ends, then were aligned to the human genome (GRCh38) using *bowtie* (v1.0.1)^61,62^. During alignment, reads were required to have a minimum length of 18 nucleotides, exhibit at most one mismatch, and align to 10 or fewer locations. After filtering, up to five best alignments were reported for each read. Aligned reads were then mapped to primary miRNA transcripts from miRBase (v. 21, 2813 miRNAs) using *bedtools* (v. 2.21.0)^63,64^. MiR-486 was greatly enriched in the final mapped reads; we removed this miR from the final analysis set as this is a reported artifact of the Illumina library prep^65,66^. We retained 247 miRNAs for analysis that were expressed at a level of ≥1 rpm in at least 20 samples. This conservative expression level cutoff maximized the correlation and performance of each of seven pairs of technical replicates included in the study. Out of 155 eligible study samples that were sequenced, one was removed for poor alignment and one was removed for having extremely high levels of rRNA, yielding a final analysis dataset of 153 samples (77 cases, 76 controls).

#### TRILOGY-ACS targeted rt-PCR

Total RNA was extracted from platelet-poor plasma samples using the Qiagen miRNAeasy Serum/Plasma kit (Venlo, NL) with spike-in control added. Following extraction, cDNA was acquired using 1.5 µl of total extracted RNA using the Qiagen miScript II RT kit. Custom miRNA PCR arrays were designed through Qiagen. The Qiagen miScript SYBR Green PCR kit was used to run the PCR reaction on a Viia 7 Real-Time PCR system (Thermo Fisher Scientific, Waltham, MA). Data were exported using an automatic baseline and cycle threshold (Ct) threshold of 0.02. A total of 46 test miRNAs were assayed in two batches, in addition to a selection of miRNAs for potential use in normalization. In each batch, samples with >25% missing data or poor assay performance as measured by Qiagen controls were removed (n = 23, 1.0%) removed from each batch. Ct values ≥ 35 were considered to be below the lower limit of quantification (LLOQ) and were designated as “undetected” measurements, while Ct values that were missing due to lack of amplification or other technical assay issues were left as missing (0.62% of all measurements). Stably-expressed endogenous miRNAs were assessed and in each batch separately using NormFinder (MOMA, Aarhus University Hospital, Denmark), and the geometric mean of the three most stable control miRNAs in each batch was used to normalize Ct measurements from the test miRNAs^67^. Ct values were then standardized for each individual miRNA to have mean=0 and standard deviation = 1 across all samples. The final analysis set contained expression measurements from 1556 samples from 878 study subjects that passed QC in both batches (655 samples from baseline, 466 from 30 days after study enrollment, and 435 from six months after study enrollment).

#### Independent Singapore cohort

The miRNeasy Kit (Qiagen) was used to isolate miRNAs from platelet-poor plasma samples. The nCounter Human miRNA Panel v2 (Nanostring, Seattle, WA) was then used to evaluate the concentration of ~800 miRNAs in these samples. MiRNA ligation and hybridization to fluorescent probes was performed at 65 °C for 18 hours, followed by probe purification and counting on the nCounter prep station and digital analyzer. MiRNA counts were thus obtained based on nCounter counts of individual fluorescent barcodes unique to individual miRNA species.

### Statistical analysis

#### Non-targeted miRNA sequencing

MiR-Seq data were analyzed using the *DESeq. 2* package (v1.18.1) in *R* (v3.4.2), which employs a negative binomial generalized linear model for differential expression analysis^68,69^. Our analysis was adjusted for potential confounders of circulating miRNA concentrations including sex, age, race, treatment arm, baseline BMI, prior clopidogrel use, aspirin and heparin status prior to enrollment, smoking status, diabetes and *CYP2C19* genotype (a known effector of P2Y_12_ metabolism) and an indicator variable to indicate whether a subject had a recurrent CV event after enrollment. Baseline PRU measurements were standardized, then tested for association with differential miRNA expression using a nominal p-value cutoff of 0.05. A p-value of <0.05 was considered statistically significant and was reported. Fold changes were Log_2_ Fold Changes calculated as the increase or decrease in miRNA expression per standard deviation in PRU. Statistical analyses were performed using SAS 9.4 (SAS Institute Inc., www.SAS.com) and R version 3.6 (The R Foundation for Statistical Computing, www.r-project.org).

#### Targeted rt-PCR

An initial analysis of the targeted plasma rt-PCR data using generalized estimating equations (GEE) was performed using a combined 30 days and six months post-enrollment cohort. This combined 30 day and 6-month analysis was utilized to improve study power and to focus our analysis to when miRNA levels and PRU levels are likely stable following a NSTE-ACS event, as inflammatory markers and associated miRNAs are known to be hyper-dynamic in the days following an NSTE-ACS event. MiRNAs with >25% undetected values (expression < LLOQ) were analyzed as binary variables (detected vs. undetected, n = 9 miRNAs), miRNAs with 10-25% undetected were analyzed as both binary and quantitative (n = 5), and miRNAs with <10% missing were considered quantitative (n = 33). GEE models were adjusted for treatment cohort and utilized sandwich variance estimates. Nominally significant plasma miRNAs (P < 0.05) from the GEE analysis were then assessed for association with PR at individual time-points using multivariable linear or logistic regression analysis, adjusted for the same clinical covariates considered in the miR-Seq analysis. Of note, miR-345-5p which was significant in the non-targeted analysis was included in the targeted analysis however it was only analyzed using logistic regression and not GEE as it had >25% missing PR values at the combined 30-day and six-month time-points. P-values from all statistical tests represent nominal values without correction for multiple comparisons. A p-value of <0.05 was considered statistically significant and was reported. Statistical analyses were performed using SAS 9.4 (SAS Institute Inc., www.SAS.com) and R version 3.6 (The R Foundation for Statistical Computing, www.r-project.org).

#### Independent Singapore cohort

MiRNA values were normalized using trimmed mean of M-values (TMM) and modeled using a negative binomial distribution using the edgeR package (version 2.4.0). Fold Change between good and poor clopidogrel responders were compared and ranked in order of significance. Differentially expressed miRNAs from the Singapore cohorts A and B were then compared to on-treatment platelet reactivity (high or low) using an exact significance (p-value) test. A p-value of <0.05 in either cohort A or B was considered statistically significant and was reported.

### Disclaimers

After reviewing the journal’s policy, the authors of this manuscript have no disclaimers to share.

## Supplementary information

Supplementary information.
