# Supplementary material for: Circulating MicroRNA Profiling in Non-ST Elevated Coronary Artery Syndrome Highlights Genomic Associations with Serial Platelet Reactivity Measurements
Source: Sci Rep. 2020 Apr 10;10:6169. doi: 10.1038/s41598-020-63263-6 (PMC7148370; doi:10.1038/s41598-020-63263-6)
Supplement: Supplementary file 1 — Supplementary information. [file 41598_2020_63263_MOESM1_ESM.pdf]

Supplemental Materials

Circulating MicroRNA Profiling in Non-ST Elevated Coronary Artery Syndrome Highlight Genomic Associations with Serial Platelet Reactivity Measurements

Kristian C. Becker[1], Lydia Coulter Kwee[1], Megan L. Neely[2], Elizabeth Grass[1], Joseph A. Jakubowski[4], Keith A.A. Fox[5], Harvey D. White[6], Simon G. Gregory[1,3], Paul A. Gurbel[7], Leonardo de Pinto Carvalho[8], Richard C. Becker[9], E. Magnus Ohman[2,3], Matthew Roe[2,3], Svati H. Shah[1,2,3]*, Mark Y. Chan[10]*

*these 2 authors supervised the work equally as senior authors

1. Duke Molecular Physiology Institute, Durham NC.
2. Duke Clinical Research Institute, Durham NC;
3. Division of Cardiology, Duke University School of Medicine, Durham NC;
4. Eli Lilly and Company, Indianapolis IN;
5. University of Edinburgh, Edinburgh, UK;
6. Green Lane Cardiovascular Service, Auckland City Hospital, Auckland, New Zealand;
7. Inova Heart & Vascular Institute, Falls Church VA;
8. Albert Einstein Hospital, Sao Paolo, Brazil
9. University of Cincinnati, Cincinnati, Ohio
10. National University of Singapore, Singapore, Singapore

Supplemental Figures: 0, Supplemental Tables: 5

Addresses for Correspondence:

Svati H. Shah, MD MHS, Duke Molecular Physiology Institute, 300 North Duke Street, Durham NC 27701, USA, Phone: (919) 684-1808, Email: svati.shah@dm.duke.edu

Mark Y. Chan, MBBS PhD, National University Heart Centre, 1E Kent Ridge Road, Singapore 119228, Singapore, Tel: +65 67725596, Fax: +65 68722998, Email: mark_chan@nuhs.edu.sg

Supplemental Materials and Methods

TRILOGY-ACS Cohort The primary outcome of the TRILOGY-ACS trial was a composite of cardiovascular events including cardiac death, MI and stroke at 36 months [70, 71]. Randomization occurred on average after 108 hours (IQR: 64, 155) of the initial cardiovascular event, specifically a NSTE-ACS event. Patients that were not able to be randomized to treatment within 72 hours of the primary event were first given a standard dose of open label clopidogrel prior to randomization [71]. Patients from the TRILOGY-ACS primary trial that were additionally enrolled in the secondary platelet function sub-study (N=2564) also provided whole blood samples at baseline, after 30 days and after six months of clopidogrel or prasugrel medication administration with simultaneous measurement of platelet reactivity (PR) at each time point [6]. PR was quantified at each individual trial site using a calibrated VerifyNow $P2Y_{12}$ Assay (Accumetrics Inc.). VerifyNow is a whole blood, adenosine diphosphate (ADP)/PGE_1 - based assay that measures platelet adhesion to fibrinogen-coated beads, with the secondary addition of PGE_1 increasing the test's specificity for $P2Y_{12}$ receptors [72-74].

Platelet reactivity profiling In the primary TRILOGY-ACS cohort, PR was measured at each participating site using the VerifyNow device $P2Y_{12}$ Assay (Accumetrics Inc), a whole blood, adenosine diphosphate (ADP) - based assay that measures platelet agglutination to fibrinogen-coated polystyrene beads. The addition of prostaglandin E_1 during the test reaction increases specificity of the test for $P2Y_{12}$ receptors. Test results are expressed as $P2Y_{12}$ reaction units (PRUs) and lower PRU readings reflected increased inhibition of the $P2Y_{12}$ receptor. PRU values were excluded if platelet adhesion measurements were performed within seven days of other anti-platelet medication administration (glycoprotein IIb/IIIa inhibitor therapy), if PRU values were determined at fewer than 10 minutes or greater than four hours after sample collection, if reported platelet inhibition was measured at greater than 100%, or if PRU values were greater > 500 [6].

In Singapore cohort A, the Vasodilator-Stimulated Phosphoprotein (VASP) flow cytometry assay (Diagnostica Stago, Asnières, France) was utilized to measure platelet sensitivity to $P2Y_{12}$ agonists and was completed within 48 hours of sample collection [40]. Citrated samples were incubated with prostaglandin E1 (PGE1) and 10 µmol/l adenosine diphosphate (ADP) for 10 min and set to plates with paraformaldehyde, and platelets were permeabilized with non-ionic detergent. VASP Analysis was performed on a FACS Canto II flow cytometer (Becton Dickinson, NJ, USA). The platelet population was identified via forward and side flow cytometry distribution and 5000 platelets were gated. We then calculated Platelet reactivity index (PRI) from median fluorescence intensity (MFI) after samples were inoculated with PGE1 or PGE1 and ADP according to the formula:

$$\mathbf{PRI = (MFI_{(PGE1)} - MFI_{(PGE1 + ADP)}/MFI_{(PGE1)}) \times 100}$$

In both Singapore cohorts A and B, PR measurements were performed by whole blood impedance aggregometry on a Multiplate analyzer (Roche Diagnostics, Basel, Switzerland) without prostaglandin addition. Experimental technique consistency across study sites was ensured by conducting centralized training of those who conducted PR testing with only one trained person at each study site conducting the testing. VASP testing was performed on citrated whole blood within 48 h of collection. All Multiplate testing was performed on whole blood samples within 1 hour of collection during cardiac catheterization.

MiRNA sequencing (miRNA-seq) from whole blood. Total RNA was extracted from PaxGENE tubes using the PerfectPure RNA blood kit (5Prime, Gaithersburg, MD) with microRNA libraries prepared using TruSeq sample prep kits (Illumina). Prior to miRNA library pooling, a Bioanalyzer DNA1000 chip (Agilent) was used for library size validation and then computed using the KAPA Library Quantification kit. Library pools were comprised of 24 libraries. MicroRNA pooled-library sequencing was performed on an Illumina HiSeq2500 as

single-end 50bp sequence runs using rapid run flow cells. MiRNA clusters were also produced for single read flow cell. Raw miRNA sequence reads were then processed using c*utadapt* v1.5 to remove Illumina sequencing adapters and low quality 3' sequence ends and aligned to the human genome (GRCh38) using *bowtie* (*version 1.0.1*) [61, 62]. 1423 miRNAs were detected with ≥0.1 mapped reads/million aligned reads (rpm) in at least one sample. During alignment, reads required a minimum length of 18 NT and no more than one mismatch per read and < 6 alignments. Reads that mapped to > 10 locations were rejected. Aligned reads were then mapped to primary miRNA transcripts through miRBase (v. 21, 2813 miRNAs) using *bedtools* (v. 2.21.0) [64, 75]. A false enrichment of miR-486 was seen in the final mapped reads, however this is a known artifact of the Illumina library prep, which has previously shown enrichment of miR-486 in excess of 50x [65]. Therefore, reads mapping to miR-486 were removed. Overall, after applying a final cutoff of ≥ 1 rpm in 16 paired samples, 247 microRNAs were analyzed. From the 20 paired samples: one pair was removed for poor alignment, with an additional two pairs removed for having high levels of rRNA, and one was removed due to poor correspondence with its technical replicate.

Targeted miRNA profiling from plasma. MiRNA enriched total RNA was extracted from plasma samples using the Qiagen miRNAeasy Serum/Plasma kit. All samples had a miRNeasy Serum/Plasma Spike-in Control added. Following extraction, cDNA was acquired using 1.5µl of total RNA extracted from plasma in the Qiagen miScript II RT kit, 200 µL of water was added to the reaction volume after reverse-transcription reaction to dilute cDNA before storage at -20$_o$C. Custom miRNA arrays were designed using miRNA assays from Qiagen; specifically, the targeted array consisted of 46 miRNAs (Suppl. Table 1), chosen based on high concentration miRNAs associated with recurrent CVD event case-control status from the miRNA-seq analysis (N=35) as well as miRNAs determined to have association with potential CVD phenotypes from the literature (N=11) (Suppl. Table 2). Each array plate had 48 miRNA assays lyophilized into

the plate and repeated eight times. Three Qiagen control assays were added to the custom array, specifically: miRTC, our targeted array specific control miRNA (miR-30e-5p, miR-30d-5p, miR-23a-3p and SNORD61), cel-miR-39 miScript and the PPC assay. The Qiagen miScript SYBR Green PCR kit was used to run the PCR reaction for the array. Each PCR was performed on a Viia 7 Real-Time PCR system. For miRNA concentration normalization plasma samples were supplemented with a *C. elegans* miR-39 miRNA mimic. An automatic baseline was used with a Ct threshold of 0.02 and Ct values normalized to cel-miR-39* for Ct ≤ 35. Ct ≥ 35 were considered as being below the lower limit of quantification. Data below LLOQ for the remaining miRNAs was imputed using the minimum concentration values minus 10%. For the Singapore cohorts, the miRNeasy Kit (Qiagen) was used to isolate miRNAs from plasma samples. The nCounter Human miRNA Panel v2 (Nanostring) was then used to evaluate the concentration of ~800 miRNAs in these samples. MiRNA ligation and hybridization to fluorescent probes was performed at 65ºC for 18 hours, followed by probe purification and counting on the nCounter prep station and digital analyzer. Data from the nCounter analyzer contained individual fluorescent barcodes that mapped to individual miRNA species and allowed for an exact count of miRNAs present in the sample.

Supplemental Figures and Tables

Supplemental Table 1: 46 miRNA Species within the Targeted qRT-PCR Array Panel

Let-7a-5p	miR-1304-3p	miR-191-5p	miR-24-3p	miR-574-3p	
Let-7d-3p	miR-1307-5p	miR-192-5p	miR-25-3p/5p	miR-636	
Let-7g-5p	miR-15b-5p	miR-20a-5p	miR-29a-3p	miR-6087	

miR-1-3p*	miR-150-5p	miR-20b-5p	miR-29c-3p*	miR-92a-3p	
miR-126-3p	miR-151a-3p	miR-208a-3p*	miR-296-5p	miR-92b-3p	
miR-126-5p	miR-17-3p*	miR-21-5p	miR-30a-5p	miR-345-5p	
miR-133a-3p	miR-18a-5p	miR-22-3p	miR-484	miR-197-3p	
miR-133b*	miR-181b-5p	miR-222-3p	miR-4685-3p*	miR-324-5p	
miR-134-5p	miR-19b-3p	miR-223-3p	miR-4746-5p	miR-939-5p	

* 6 miRNA Species were excluded from the analysis following Internal Quality Control measures

Supplemental Table 2: Detailed reasoning for miRNA chosen for targeted qRT-PCR array.

MicroRNA	Involvement in Platelet/CVD [Reference]
Let-7a, Let-7d, Let-7g	• Part of Let-7 family, abundant in platelets
miR-1-3p	• Known platelet enriched miR<br>• Highly cited in cardiac hypertrophy [76]
miR-126	• Athero-protective effects of endothelial apoptotic bodies<br>• Known controller of angiogenesis [77]
miR-1304-3p	• Associated with lowering BNP and type I/II MI
miR-1307-5p	• miR-1307-5p; predictive of angiogenic response in medication [78]<br>• Highly expressed in TRILOGY patients with high BNP.
miR-133a-3p	• Part of miR-1/133 cluster, many publications on role in cardiac hypertrophy and remodeling [79]<br>• Considered a key biomarker in heart failure [80]

miR-133b	• Differentially expressed in sample patients on clopidogrel vs prasugrel at 30 days.<br>• Potential biomarker in acute MI [81]
miR-134	• Reported to be circulating in Acute MI [82]
miR-150-5p	• Prognostic of ventricular remodeling and HF events [83]
miR-151a-3p	• Down regulated in experimental models of heart failure, increased susceptibility to post-MI arrhythmias [78]
miR-15b-5p	• MiR-15 family implicated in ventricular hypertrophy and modulation of infarct size [84]
miR-17-3p*	• Part of the miR-17/miR-92 supercluster [85]<br>• Regulatory unit in ischemia/reperfusion injury [86]<br>• Mir-17-5p but not miR-17-3p assessed in the Nanostring miRNA set
miR-181b-5p	• Down regulated in in response to transverse aortic constriction, implicated in cardiac hypertrophy via NF-κB [87]
miR-18a-5p	• miR-18a-5p; Part of the miR-17/miR-92 super cluster.
miR-191-5p	• Platelet enriched miRNA [49]
miR-192-5p	• A circulating p53-responsive microRNA predictive indicators of heart failure after acute MI [88]
miR-19b-3p	• Altered at baseline in miRNA-Seq (p=0.05).
miR-208a-3p	• Strongly highlighted as potential ACS biomarker [89]
miR-20a-5p	• Part of the miR-17/miR-92 super cluster.
miR-20b-5p	• Platelet enriched miR (anti-angiogenic). Responds differentially to antiplatelet therapy of different potency [90]
miR-21-5p	• Released from cardiac fibroblasts (pro-apoptotic) and induced in failing heart [91]
miR-22-3p	• Overexpression in cardiomyocyte causing hypertrophy [92]
miR-222-3p	• Inhibits endothelial cell migration, contributes to sex-dimorphic eNOS expression via ETS-1 [93]
miR-223-3p	• miR-223-3p; Platelet enriched miR, often cited as being key platelet-derived miR playing role in CV outcomes [15, 94]
miR-24-3p	• Platelet enriched miR, expression induces cardiac hypertrophy [95, 96]
miR-25-3p	• Highly expressed in miRNA-Seq dataset.<br>• Inhibition improves contractility in heart failure [97]

miR-29a-3p	• Significant role of miR-29 in cardiac fibrosis following MI [98]
miR-29c-5p	• Critical role in cardiac fibrosis following MI [98]<br>• Highly expressed in miR-Seq dataset
miR-30a-5p	• Highly expressed in miRNA-Seq dataset, particularly in those with low BNP/CRP.
miR-4685-3p*	• Highly expressed in miRNA-Seq dataset.<br>• Not included in the Nanostring miRNA set
miR-4746-5p*	• Highly expressed in miRNA-Seq dataset, particularly in non-diabetic subjects.<br>• Not included in the Nanostring miRNA set
miR-484	• Inhibitor of mitochondrial function and potential predictor of spontaneous MI [99]
miR-574-3p	• Highly expressed in miRNA-seq dataset
miR-6087*	• Highly expressed in miRNA-Seq dataset<br>• Down regulates endothelial endoglin expression [100]<br>• Not included in the Nanostring miRNA set
miR-636*	• Highly expressed in miRNA-Seq dataset<br>• Early biomarker of MI in whole blood miRNA sequencing study [101]<br>• Not included in the Nanostring miRNA set
miR-92a-3p	• Highly expressed in the miRNA-Seq dataset.<br>• Member of the miR-17/miR-92 super-cluster [85]
miR-92b-3p	• Highly expressed in the miRNA-Seq dataset.

Supplemental Table 3A: Baseline characteristics of Singapore cohort A: A comparison between 24 patients with high on-treatment platelet reactivity (VASP PRI > 50%) and 24 patients with low on treatment platelet reactivity (VASP PRI < 50%).

	**HPR**	**LPR**	**P-value**
**Age**	57 (51–64)	53 (50–64.5)	0.705
**Female**	33.3	13.5	0.519
**Ethnicity**			0.132
**Chinese**	67.4	56.8	
**Malay**	20.4	32.4	

**Indian**	12.2	10.8	
**Diabetes**	34.7	48.7	0.426
**BMI (kg/m2)**	25 (23, 27)	26 (24, 28.5)	0.371
**Serum creatinine (μmol/L)**	75 (67.5, 85)	84.5 (70.5, 99.25)	0.07
**Coronary artery disease†**	91.8	86.4	0.945
**Proton pump inhibitor**	30.6	29.7	1
**DHP calcium channel antagonist**	26.9	32.5	0.893
**Statin**	66.7	78.4	0.461
**Aspirin**	100	100	1
**Smoking**	34.7	32.4	0.659

* Continuous variables are presented as median (25th and 75th percentile) and categorical variables are presented as percentages

Supplemental Table 3B: Baseline characteristics of Singapore cohort B: A comparison between 24 patients with high on-treatment platelet reactivity (ADP > 46 aggregation units) and 24 patients with low on treatment platelet reactivity (ADP < 46 aggregation units).

	**HPR (N=24)**	**LPR (N=24)**	**P-value**
**Age (years)**	56 (47, 60)	56 (50,63)	0.27
**Men (%)**	95	95	0.74
**Race**			0.08
**Chinese (%)**	50	85	
**Malay (%)**	14	5	
**Indian (%)**	36	10	
**Body-Mass Index (kg/m2)**	24.3 (23.4-27.1)	24.2 (23.4-26.8)	0.68
**Hypertension (%)**	67	63	0.08
**Dyslipidemia (%)**	68.2	52.4	0.36
**Current Smoker (%)**	40.9	33.3	0.70
**Diabetes (%)**	37	35	0.46
**GRACE Score**	102 (79-112)	86 (79-98)	0.11
**NSTE-ACS (%)**	82	79	0.16
**ADP test result (AU*min)**	926 (765-1191)	210 (185-226)	<0.01

**Initial serum creatinine value (µmol/dL)**	85 (67-101)	75 (68-84)	0.24
**Baseline platelet count ($\times 10^3$ cells per mm^3)**	254 (212-316)	210 (204-234)	<0.01

Continuous variables expressed as median (25th, 75th percentile) HPR = High on-ADP receptor antagonist platelet reactivity (ADP test ≥ 468 AU*min). LPR= Low on-ADP receptor antagonist platelet reactivity (ADP test < 468 AU*min)

Supplemental Table 4. MiRNAs significantly associated with platelet reactivity in the Singapore cohort in baseline plasma samples. Fold Changes represent Log_2 Fold Changes in miRNA concentration per 1 SD PRU unit. MiRNA species are listed from smallest to largest p-value in cohort A. MiRNA species significant in either cohort A or B were included. MiRNA effect sizes (Fold Changes) ranged from -0.279 to 0.630 with p-values from $1.6x10^{-13}$ to 0.18. § MiRNA species that were also significantly associated with PRU in the TRILOGY cohort.

MiRNA	Cohort A Log_2 Fold Change (P-value)	Cohort B Log_2 Fold Change (P-value)
Let-7a-5p	0.24 (0.033)	0.50 ($1.3x10^{-12}$)
Let-7b-5p	0.30 ($7.3x10^{-4}$)	0.35 ($3.1x10^{-7}$)
miR-106a-5p	0.21 (0.024)	0.24 ($4.5x10^{-4}$)
miR-126-3p§	0.33 (0.009)	0.45 ($3.2x10^{-11}$)
miR-142-3p	0.17 (0.091)	0.29 ($1.1x10^{-5}$)
miR-142-5p	-0.18 (0.07)	-0.1 (0.02)
miR-146a-5p	0.18 (0.07)	0.44 ($1.4x10^{-10}$)
miR-155-5p	-0.25 (0.01)	-0.44 ($7.2x10^{-8}$)
miR-15b-5p§	0.2 (0.06)	0.46 ($2.0x10^{-11}$)

miR-17-5p	0.21 (0.02)	0.24 ($4.5x10^{-4}$)
miR-181a-5p	0.34 ($2.4x10^{-3}$)	0.38 ($5.2x10^{-8}$)
miR-1912	-0.29 (0.01)	-0.1 (0.03)
miR-19b-3p	0.29 ($2.0x10^{-3}$)	0.21 ($2.1x10^{-3}$)
miR-20a-5p/miR-20b-5p	0.28 ($2.4x10^{-3}$)	0.25 ($2.6x10^{-4}$)
miR-223-3p	0.52 ($1.4x10^{-9}$)	0.27 ($3.8x10^{-5}$)
miR-23a-3p	0.38 ($9.1x10^{-5}$)	0.16 (0.013)
miR-361-3p	0.24 (0.074)	0.16 (0.045)
miR-4454	0.37 ($1.5x10^{-5}$)	0.32 ($8.5x10^{-7}$)
miR-451a	0.63 ($1.6x10^{-13}$)	0.16 (0.023)
miR-576-5p	-0.16 (0.16)	-0.1 (0.024)
miR-601	-0.13 (0.18)	-0.31 ($6x10^{-6}$)
miR-720	0.24 ($4.2x10^{-3}$)	0.28 ($5.4x10^{-5}$)
miR-92a-3p	0.34 ($3.4x10^{-4}$)	0.25 ($2.8x10^{-4}$)
miR-93-5p§	0.23 (0.01)	0.24 ($4.0x10^{-4}$)

Supplemental References:

1. Bray, P.F., *Platelet reactivity and genetics down on the pharm.* Trans Am Clin Climatol Assoc, 2006. **117**: p. 103-11; discussion 111-2.
2. Camaioni, C., et al., *Microparticles and microRNAs: new players in the complex field of coagulation.* Intern Emerg Med, 2013. **8**(4): p. 291-6.
3. Go, A.S., et al., *Heart disease and stroke statistics--2014 update: a report from the American Heart Association.* Circulation, 2014. **129**(3): p. e28-e292.
4. Roffi, M., et al., *2015 ESC Guidelines for the management of acute coronary syndromes in patients presenting without persistent ST-segment elevation: Task Force for the Management of Acute Coronary Syndromes in Patients Presenting without Persistent ST-Segment Elevation of the European Society of Cardiology (ESC).* Eur Heart J, 2016. **37**(3): p. 267-315.

5. Angiolillo, D.J., et al., *Impact of platelet reactivity on cardiovascular outcomes in patients with type 2 diabetes mellitus and coronary artery disease.* J Am Coll Cardiol, 2007. **50**(16): p. 1541-7.
6. Gurbel, P.A., et al., *Platelet function during extended prasugrel and clopidogrel therapy for patients with ACS treated without revascularization: the TRILOGY ACS platelet function substudy.* Jama, 2012. **308**(17): p. 1785-94.
7. Gurbel, P.A., et al., *Platelet reactivity in patients and recurrent events post-stenting: results of the PREPARE POST-STENTING Study.* J Am Coll Cardiol, 2005. **46**(10): p. 1820-6.
8. Tantry, U.S., et al., *Consensus and update on the definition of on-treatment platelet reactivity to adenosine diphosphate associated with ischemia and bleeding.* J Am Coll Cardiol, 2013. **62**(24): p. 2261-73.
9. Shuldiner, A.R., et al., *Association of cytochrome P450 2C19 genotype with the antiplatelet effect and clinical efficacy of clopidogrel therapy.* JAMA, 2009. **302**(8): p. 849-57.
10. Varenhorst, C., et al., *Effect of genetic variations on ticagrelor plasma levels and clinical outcomes.* Eur Heart J, 2015. **36**(29): p. 1901-12.
11. Karazniewicz-Lada, M., et al., *Impact of common ABCB1 polymorphism on pharmacokinetics and pharmacodynamics of clopidogrel and its metabolites.* J Clin Pharm Ther, 2015. **40**(2): p. 226-31.
12. Yamaguchi, Y., et al., *Effects of VerifyNow P2Y12 test and CYP2C19*2 testing on clinical outcomes of patients with cardiovascular disease: a systematic review and meta-analysis.* Platelets, 2013. **24**(5): p. 352-61.
13. Lewis, J.P., et al., *Genetic variation in PEAR1 is associated with platelet aggregation and cardiovascular outcomes.* Circ Cardiovasc Genet, 2013. **6**(2): p. 184-92.
14. Bozzi, L.M., et al., *The Pharmacogenomics of Anti-Platelet Intervention (PAPI) Study: Variation in Platelet Response to Clopidogrel and Aspirin.* Curr Vasc Pharmacol, 2016. **14**(1): p. 116-24.
15. Kaudewitz, D., et al., *Association of MicroRNAs and YRNAs With Platelet Function.* Circ Res, 2016. **118**(3): p. 420-32.
16. Cavarretta, E., G.A. Chiariello, and G. Condorelli, *Platelets, endothelium, and circulating microRNA-126 as a prognostic biomarker in cardiovascular diseases: per aspirin ad astra.* Eur Heart J, 2013. **34**(44): p. 3400-2.
17. Gatsiou, A., et al., *MicroRNAs in platelet biogenesis and function: implications in vascular homeostasis and inflammation.* Curr Vasc Pharmacol, 2012. **10**(5): p. 524-31.
18. Olson, E.N., *MicroRNAs as therapeutic targets and biomarkers of cardiovascular disease.* Sci Transl Med, 2014. **6**(239): p. 239ps3.
19. Shi, R., et al., *Decreased platelet miR-223 expression is associated with high on-clopidogrel platelet reactivity.* Thromb Res, 2013. **131**(6): p. 508-13.
20. Kuwabara, Y., et al., *Increased microRNA-1 and microRNA-133a levels in serum of patients with cardiovascular disease indicate myocardial damage.* Circ Cardiovasc Genet, 2011. **4**(4): p. 446-54.
21. Karakas, M., et al., *Circulating microRNAs strongly predict cardiovascular death in patients with coronary artery disease-results from the large AtheroGene study.* Eur Heart J, 2016.

22. Wiviott, S.D., et al., *Prasugrel compared with high loading- and maintenance-dose clopidogrel in patients with planned percutaneous coronary intervention: the Prasugrel in Comparison to Clopidogrel for Inhibition of Platelet Activation and Aggregation-Thrombolysis in Myocardial Infarction 44 trial.* Circulation, 2007. **116**(25): p. 2923-32.
23. D'Alessandra, Y., et al., *Circulating microRNAs are new and sensitive biomarkers of myocardial infarction.* Eur Heart J, 2010. **31**(22): p. 2765-73.
24. Freitas, R.C.C., et al., *Integrated analysis of miRNA and mRNA gene expression microarrays: Influence on platelet reactivity, clopidogrel response and drug-induced toxicity.* Gene, 2016. **593**(1): p. 172-178.
25. Kim, E.Y., et al., *SENP5, a SUMO isopeptidase, induces apoptosis and cardiomyopathy.* J Mol Cell Cardiol, 2015. **78**: p. 154-64.
26. Shimizu, Y., et al., *DJ-1 protects the heart against ischemia-reperfusion injury by regulating mitochondrial fission.* J Mol Cell Cardiol, 2016. **97**: p. 56-66.
27. Tonge, D.P. and T.W. Gant, *What is normal? Next generation sequencing-driven analysis of the human circulating miRNAOme.* BMC Mol Biol, 2016. **17**: p. 4.
28. Witkowski, M., et al., *Micro-RNA-126 Reduces the Blood Thrombogenicity in Diabetes Mellitus via Targeting of Tissue Factor.* Arterioscler Thromb Vasc Biol, 2016. **36**(6): p. 1263-71.
29. Schober, A., et al., *MicroRNA-126-5p promotes endothelial proliferation and limits atherosclerosis by suppressing Dlk1.* Nat Med, 2014. **20**(4): p. 368-76.
30. Li, H.Y., et al., *Plasma MicroRNA-126-5p is Associated with the Complexity and Severity of Coronary Artery Disease in Patients with Stable Angina Pectoris.* Cell Physiol Biochem, 2016. **39**(3): p. 837-46.
31. Garcia, A., et al., *Functional Validation of microRNA-126-3p as a Platelet Reactivity Regulator Using Human Haematopoietic Stem Cells.* Thromb Haemost, 2019. **119**(2): p. 254-263.
32. Laffont, B., et al., *Platelet microparticles reprogram macrophage gene expression and function.* Thromb Haemost, 2016. **115**(2): p. 311-23.
33. Sapp, R.M., et al., *Circulating microRNAs and endothelial cell migration rate are associated with metabolic syndrome and fitness level in postmenopausal African American women.* Physiol Rep, 2019. **7**(14): p. e14173.
34. Wang, Y., et al., *Predictive value of circulating coagulation related microRNAs expressions for major adverse cardiac and cerebral event risk in patients undergoing continuous ambulatory peritoneal dialysis: a cohort study.* J Nephrol, 2019.
35. Codagnone, M., et al., *Lipoxin A4 stimulates endothelial miR-126-5p expression and its transfer via microvesicles.* FASEB J, 2017. **31**(5): p. 1856-1866.
36. Ayaz, L. and E. Dinc, *Evaluation of microRNA responses in ARPE-19 cells against the oxidative stress.* Cutan Ocul Toxicol, 2017: p. 1-6.
37. Ma, W., et al., *Identification of microRNAs involved in gefitinib resistance of non-small-cell lung cancer through the insulin-like growth factor receptor 1 signaling pathway.* Exp Ther Med, 2017. **14**(4): p. 2853-2862.
38. Hers, I., *Insulin-like growth factor-1 potentiates platelet activation via the IRS/PI3Kalpha pathway.* Blood, 2007. **110**(13): p. 4243-52.
39. Wang, X., et al., *Mitochondria Associated MicroRNA Expression Profiling of Heart Failure.* Biomed Res Int, 2017. **2017**: p. 4042509.

40. Wei, Q., et al., *MiR-345-3p attenuates apoptosis and inflammation caused by oxidized low-density lipoprotein by targeting TRAF6 via TAK1/p38/NF-kB signaling in endothelial cells.* Life Sci, 2019. **241**: p. 117142.
41. Yu, M.L., et al., *Vascular smooth muscle cell proliferation is influenced by let-7d microRNA and its interaction with KRAS.* Circ J, 2011. **75**(3): p. 703-9.
42. Wang, Y., et al., *Let-7d miRNA prevents TGF-beta1-induced EMT and renal fibrogenesis through regulation of HMGA2 expression.* Biochem Biophys Res Commun, 2016. **479**(4): p. 676-682.
43. Doerr, M., et al., *Differential effect of hypoxia on early endothelial-mesenchymal transition response to transforming growth beta isoforms 1 and 2.* Microvasc Res, 2016. **108**: p. 48-63.
44. Baldeon Rojas, L., et al., *Study on inflammation-related genes and microRNAs, with special emphasis on the vascular repair factor HGF and miR-574-3p, in monocytes and serum of patients with T2D.* Diabetol Metab Syndr, 2016. **8**: p. 6.
45. Zhou, J., et al., *miRNA 206 and miRNA 574-5p are highly expression in coronary artery disease.* Biosci Rep, 2016. **36**(1): p. e00295.
46. Lai, Z., et al., *MicroRNA-574-5p promotes cell growth of vascular smooth muscle cells in the progression of coronary artery disease.* Biomed Pharmacother, 2018. **97**: p. 162-167.
47. Corsten, M.F., et al., *Circulating MicroRNA-208b and MicroRNA-499 reflect myocardial damage in cardiovascular disease.* Circ Cardiovasc Genet, 2010. **3**(6): p. 499-506.
48. Dong, H., et al., *Trace and label-free microRNA detection using oligonucleotide encapsulated silver nanoclusters as probes.* Anal Chem, 2012. **84**(20): p. 8670-4.
49. Willeit, P., et al., *Circulating microRNAs as novel biomarkers for platelet activation.* Circ Res, 2013. **112**(4): p. 595-600.
50. Kaudewitz, D., et al., *Impact of intravenous heparin on quantification of circulating microRNAs in patients with coronary artery disease.* Thromb Haemost, 2013. **110**(3): p. 609-15.
51. Breet, N.J., et al., *Comparison of platelet function tests in predicting clinical outcome in patients undergoing coronary stent implantation.* JAMA, 2010. **303**(8): p. 754-62.
52. Aradi, D., et al., *Efficacy and safety of intensified antiplatelet therapy on the basis of platelet reactivity testing in patients after percutaneous coronary intervention: systematic review and meta-analysis.* Int J Cardiol, 2013. **167**(5): p. 2140-8.
53. Aradi, D., et al., *Expert position paper on the role of platelet function testing in patients undergoing percutaneous coronary intervention.* Eur Heart J, 2014. **35**(4): p. 209-15.
54. Jakob, P., et al., *Profiling and validation of circulating microRNAs for cardiovascular events in patients presenting with ST-segment elevation myocardial infarction.* Eur Heart J, 2017. **38**(7): p. 511-515.
55. Naga Prasad, S.V., et al., *A unique microRNA profile in end-stage heart failure indicates alterations in specific cardiovascular signaling networks.* PLoS One, 2017. **12**(3): p. e0170456.
56. Viereck, J. and T. Thum, *Circulating Noncoding RNAs as Biomarkers of Cardiovascular Disease and Injury.* Circ Res, 2017. **120**(2): p. 381-399.
57. Sunderland, N., et al., *MicroRNA Biomarkers and Platelet Reactivity: The Clot Thickens.* Circ Res, 2017. **120**(2): p. 418-435.

58. Roe, M.T., et al., *Prasugrel versus clopidogrel for acute coronary syndromes without revascularization.* N Engl J Med, 2012. **367**(14): p. 1297-309.
59. Sen, H.M., et al., *Effects of CYP2C19 and P2Y12 Gene Polymorphisms on Clinical Results of Patients Using Clopidogrel after Acute Ischemic Cerebrovascular Disease.* Balkan J Med Genet, 2014. **17**(2): p. 37-41.
60. de Carvalho, L.P., et al., *Prognostic Implications of Dual Platelet Reactivity Testing in Acute Coronary Syndrome.* Thromb Haemost, 2018. **118**(2): p. 415-426.
61. Martin, M., *Cutadapt removes adapter sequences from high-throughput sequencing reads.* EMBnet.journal, 2012. **17**.
62. Langmead, B., et al., *Ultrafast and memory-efficient alignment of short DNA sequences to the human genome.* Genome Biol, 2009. **10**(3): p. R25.
63. Quinlan, A.R., *BEDTools: The Swiss-Army Tool for Genome Feature Analysis.* Curr Protoc Bioinformatics, 2014. **47**: p. 11 12 1-11 12 34.
64. Kozomara, A. and S. Griffiths-Jones, *miRBase: annotating high confidence microRNAs using deep sequencing data.* Nucleic Acids Res, 2014. **42**(Database issue): p. D68-73.
65. Huang, X., et al., *Characterization of human plasma-derived exosomal RNAs by deep sequencing.* BMC Genomics, 2013. **14**: p. 319.
66. Wang, A., et al., *Whole blood sequencing reveals circulating microRNA associations with high-risk traits in non-ST-segment elevation acute coronary syndrome.* Atherosclerosis, 2017. **261**: p. 19-25.
67. Andersen, C.L., J.L. Jensen, and T.F. Orntoft, *Normalization of real-time quantitative reverse transcription-PCR data: a model-based variance estimation approach to identify genes suited for normalization, applied to bladder and colon cancer data sets.* Cancer Res, 2004. **64**(15): p. 5245-50.
68. Love, M.I., W. Huber, and S. Anders, *Moderated estimation of fold change and dispersion for RNA-seq data with DESeq2.* Genome Biol, 2014. **15**(12): p. 550.
69. Seo, M., et al., *RNA-seq analysis for detecting quantitative trait-associated genes.* Sci Rep, 2016. **6**: p. 24375.
70. Chin, C.T., et al., *Study design and rationale of a comparison of prasugrel and clopidogrel in medically managed patients with unstable angina/non-ST-segment elevation myocardial infarction: the TaRgeted platelet Inhibition to cLarify the Optimal strateGy to medicallY manage Acute Coronary Syndromes (TRILOGY ACS) trial.* Am Heart J, 2010. **160**(1): p. 16-22 e1.
71. Roe, M.T., et al., *Prasugrel versus clopidogrel for acute coronary syndromes without revascularization.* N Engl J Med, 2012. **367**(14): p. 1297-309.
72. Malinin, A., et al., *Monitoring platelet inhibition after clopidogrel with the VerifyNow-P2Y12(R) rapid analyzer: the VERIfy Thrombosis risk ASsessment (VERITAS) study.* Thromb Res, 2007. **119**(3): p. 277-84.
73. Malinin, A., et al., *Validation of a VerifyNow-P2Y12 cartridge for monitoring platelet inhibition with clopidogrel.* Methods Find Exp Clin Pharmacol, 2006. **28**(5): p. 315-22.
74. von Beckerath, N., et al., *Assessment of platelet response to clopidogrel with multiple electrode aggregometry, the VerifyNow P2Y12 analyzer and platelet Vasodilator-Stimulated Phosphoprotein flow cytometry.* Blood Coagul Fibrinolysis, 2010. **21**(1): p. 46-52.

75. Quinlan, A.R., *BEDTools: The Swiss-Army Tool for Genome Feature Analysis.* Curr Protoc Bioinformatics, 2014. **47**: p. 11 12 1-34.
76. Ikeda, S., et al., *MicroRNA-1 negatively regulates expression of the hypertrophy-associated calmodulin and Mef2a genes.* Mol Cell Biol, 2009. **29**(8): p. 2193-204.
77. Zernecke, A., et al., *Delivery of microRNA-126 by apoptotic bodies induces CXCL12-dependent vascular protection.* Sci Signal, 2009. **2**(100): p. ra81.
78. Collares, C.V., et al., *Identifying common and specific microRNAs expressed in peripheral blood mononuclear cell of type 1, type 2, and gestational diabetes mellitus patients.* BMC Res Notes, 2013. **6**: p. 491.
79. Huang, L., et al., *Phenanthrene exposure induces cardiac hypertrophy via reducing miR-133a expression by DNA methylation.* Sci Rep, 2016. **6**: p. 20105.
80. Besler, C., et al., *Endomyocardial miR-133a levels correlate with myocardial inflammation, improved left ventricular function, and clinical outcome in patients with inflammatory cardiomyopathy.* Eur J Heart Fail, 2016. **18**(12): p. 1442-1451.
81. Cortez-Dias, N., et al., *Circulating miR-122-5p/miR-133b Ratio Is a Specific Early Prognostic Biomarker in Acute Myocardial Infarction.* Circ J, 2016. **80**(10): p. 2183-91.
82. He, F., et al., *Predictive value of circulating miR-328 and miR-134 for acute myocardial infarction.* Mol Cell Biochem, 2014. **394**(1-2): p. 137-44.
83. Liu, Z., et al., *MicroRNA-150 protects the heart from injury by inhibiting monocyte accumulation in a mouse model of acute myocardial infarction.* Circ Cardiovasc Genet, 2015. **8**(1): p. 11-20.
84. Liu, L.F., et al., *MicroRNA-15a/b are up-regulated in response to myocardial ischemia/reperfusion injury.* J Geriatr Cardiol, 2012. **9**(1): p. 28-32.
85. Fichtlscherer, S., et al., *Circulating microRNAs in patients with coronary artery disease.* Circ Res, 2010. **107**(5): p. 677-84.
86. Zhou, M., et al., *MiR-17-92 cluster is a novel regulatory gene of cardiac ischemic/reperfusion injury.* Med Hypotheses, 2013. **81**(1): p. 108-10.
87. Sun, X., et al., *MicroRNA-181b regulates NF-kappaB-mediated vascular inflammation.* J Clin Invest, 2012. **122**(6): p. 1973-90.
88. Matsumoto, S., et al., *Circulating p53-responsive microRNAs are predictive indicators of heart failure after acute myocardial infarction.* Circ Res, 2013. **113**(3): p. 322-6.
89. De Rosa, S., et al., *Transcoronary concentration gradients of circulating microRNAs.* Circulation, 2011. **124**(18): p. 1936-44.
90. Zampetaki, A., et al., *Plasma microRNA profiling reveals loss of endothelial miR-126 and other microRNAs in type 2 diabetes.* Circ Res, 2010. **107**(6): p. 810-7.
91. Bang, C., et al., *Cardiac fibroblast-derived microRNA passenger strand-enriched exosomes mediate cardiomyocyte hypertrophy.* J Clin Invest, 2014. **124**(5): p. 2136-46.
92. Hong, Y., et al., *MiR-22 may Suppress Fibrogenesis by Targeting TGFbetaR I in Cardiac Fibroblasts.* Cell Physiol Biochem, 2016. **40**(6): p. 1345-1353.
93. Evangelista, A.M., et al., *miR-222 contributes to sex-dimorphic cardiac eNOS expression via ets-1.* Physiol Genomics, 2013. **45**(12): p. 493-8.
94. Liu, X., et al., *MiR-223-3p as a Novel MicroRNA Regulator of Expression of Voltage-Gated K+ Channel Kv4.2 in Acute Myocardial Infarction.* Cell Physiol Biochem, 2016. **39**(1): p. 102-14.

95. Talasila, A., et al., *Myocardin regulates vascular response to injury through miR-24/-29a and platelet-derived growth factor receptor-beta.* Arterioscler Thromb Vasc Biol, 2013. **33**(10): p. 2355-65.
96. Wang, J., et al., *MicroRNA-24 regulates cardiac fibrosis after myocardial infarction.* J Cell Mol Med, 2012. **16**(9): p. 2150-60.
97. Wahlquist, C., et al., *Inhibition of miR-25 improves cardiac contractility in the failing heart.* Nature, 2014. **508**(7497): p. 531-5.
98. van Rooij, E., et al., *Dysregulation of microRNAs after myocardial infarction reveals a role of miR-29 in cardiac fibrosis.* Proc Natl Acad Sci U S A, 2008. **105**(35): p. 13027-32.
99. Wang, K., et al., *MDRL lncRNA regulates the processing of miR-484 primary transcript by targeting miR-361.* PLoS Genet, 2014. **10**(7): p. e1004467.
100. Yoo, J.K., et al., *Discovery and characterization of novel microRNAs during endothelial differentiation of human embryonic stem cells.* Stem Cells Dev, 2012. **21**(11): p. 2049-57.
101. Vogel, B., et al., *Refining diagnostic microRNA signatures by whole-miRNome kinetic analysis in acute myocardial infarction.* Clin Chem, 2013. **59**(2): p. 410-8.
